# Association of primary allostatic load mediators and metabolic syndrome (MetS): A systematic review

**DOI:** 10.3389/fendo.2022.946740

**Published:** 2022-11-22

**Authors:** Francis Osei, Andrea Block, Pia-Maria Wippert

**Affiliations:** Medical Sociology and Psychobiology, Department of Health and Physical Activity, University of Potsdam, Brandenburg, Germany

**Keywords:** allostatic load, cortisol, dehydroepiandrosterone sulfate, epinephrine, norepinephrine, metabolic syndrome, primary marker

## Abstract

Allostatic load (AL) exposure may cause detrimental effects on the neuroendocrine system, leading to metabolic syndrome (MetS). The primary mediators of AL involve serum dehydroepiandrosterone sulfate (DHEAS; a functional HPA axis antagonist); further, cortisol, urinary norepinephrine (NE), and epinephrine (EPI) excretion levels (assessed within 12-h urine as a golden standard for the evaluation of the HPA axis activity and sympathetic nervous system activity). However, the evidence of an association between the primary mediators of AL and MetS is limited. This systematic review aimed to critically examine the association between the primary mediators of AL and MetS. PubMed and Web of Science were searched for articles from January 2010 to December 2021, published in English. The search strategy focused on cross-sectional and case–control studies comprising adult participants with MetS, obesity, overweight, and without chronic diseases. The STROBE checklist was used to assess study quality control. Of 770 studies, twenty-one studies with a total sample size (*n* = 10,666) met the eligibility criteria. Eighteen studies were cross-sectional, and three were case–control studies. The included studies had a completeness of reporting score of COR % = 87.0 ± 6.4%. It is to be noted, that cortisol as a primary mediator of AL showed an association with MetS in 50% (urinary cortisol), 40% (serum cortisol), 60% (salivary cortisol), and 100% (hair cortisol) of the studies. For DHEAS, it is to conclude that 60% of the studies showed an association with MetS. In contrast, urinary EPI and urinary NE had 100% no association with MetS. In summary, there is a tendency for the association between higher serum cortisol, salivary cortisol, urinary cortisol, hair cortisol, and lower levels of DHEAS with MetS. Future studies focusing on longitudinal data are warranted for clarification and understanding of the association between the primary mediators of AL and MetS.

## 1 Introduction

Metabolic syndrome (MetS) is defined as the cluster of co-existence of high blood pressure, abdominal obesity, low high-density lipoprotein (HDL) cholesterol, elevated triglycerides, and hyperglycemia (1, 2). These metabolic abnormalities have been linked to the development of type 2 diabetes (T2DM) and cardiovascular diseases (CVDs) (3). Globally, the prevalence of MetS is estimated to affect over 20% of the adult population in the USA (4), China (5), Europe (6), as well as developing countries (7, 8). The potential causal and influencing factors of MetS may be genetic, environmental (e.g., socioeconomic status, urbanicity), psychosocial (e.g., perceived stress, depression), behavioral (e.g., physical activity), and biographical (e.g., education, childhood adversity) factors that are often conditioned by sex and age (9, 10). A current meta-analysis study that involved total patients (*n* = 162,450) reported that MetS increased adverse cardiovascular events and mortality rates (11). Similarly, a previous systematic review reported that cumulative stress termed “allostatic load (AL)” is associated with CVDs, diabetes, and MetS (12). A very well-evaluated index for the assessment of chronic stress is the AL index, which reflects the impact of chronic stress on different allosteric systems and pathways (13, 14). Allostasis is an adaptive response mechanism to chronic stress to restore physiological stability through the autonomic nervous system (ANS), the hypothalamic–pituitary–adrenal axis (HPA), the hypothalamic–pituitary–thyroid axis (HPT), somatotropic axes (i.e., growth hormones [GH], insulin-like growth factors [IGF-I and III] and their associated carrier proteins and receptors), gonadal axis (HPG), and the metabolic and immune system (15–18). Moreover, AL is the strain on the body resulting from repeated up and downregulation of physiologic stress response, as well as by the elevated activity of physiologic systems under chronic challenge, the changes in metabolism, and the impact of wear and tear on several organs and tissues that predispose the organism to disease (19, 20).

The concept of the measurement of allostasis and AL is integrated with the AL index, which was first discussed by Seeman et al. (21). Seeman et al. (21) assessed AL using 10 biomarkers. The gold standard for the evaluation of AL is the measurement of 24 biomarkers, which are summarized into an index (22) and theoretically differentiated into primary and secondary mediators of the AL index (23, 24). The primary mediators of AL consist of four biomarkers involving serum dehydroepiandrosterone sulfate (DHEAS; a functional HPA axis antagonist); 12-h urinary cortisol excretion (an integrated measure of 12-h HPA axis activity); and 12-h epinephrine (EPI) and norepinephrine (NE) excretion levels (integrated indices of 12-h sympathetic nervous system activity) (25). The remaining six biomarkers, which are considered secondary mediators of AL, overlap with the biomarkers used in the diagnosis of MetS (14). It has been shown that there is a co-activation of the HPA axis and sympathetic adrenal medullary system (SAM) under stress (26). While the HPA axis secretes glucocorticoids (e.g., cortisol), the SAM secretes catecholamines (e.g., EPI and NE). Stress can alter glucocorticoid function to enhance gluconeogenesis and free fatty acids (FFA) by differentiation of pre-adipocytes leading to central fat accumulation and MetS development (27). On the one hand, cortisol helps to regulate SAM to create optimum homeostasis when an individual encounters acute stress. On the other hand, chronic stress leads to prolonged activation of SAM and alterations in HPA axis function in the cardiovascular, metabolic, immunologic, and central nervous systems (28). Higher cortisol levels lead to obesity and MetS (29, 30). Additionally, both dehydroepiandrosterone (DHEA) and its sulfate ester DHEAS are steroid hormones connected to stress (31). Physiologically, both DHEA and DHEAS exert anti-glucocorticoid activity (32, 33), and catecholamine synthesis and secretion (34). Low DHEAS levels and an age-related decline in DHEAS may cause higher circulating cortisol in peripheral target tissues, contributing to insulin resistance, obesity, and MetS (35, 36).

Furthermore, catecholamines such as EPI and NE modulate corticotrophin-releasing hormone (CRH) and adrenocorticotropic hormone (ACTH) during both acute and chronic stress challenges (37, 38). Ebert et al. (39) revealed that psychological stress mediated the development of MetS through the release of EPI and NE. Increasing doses of catecholamines show greater lipolytic effects on visceral fats *via* the β_1_- and β_2_-adrenoceptors (40). Furthermore, Ziegler et al. (41) reported that β-adrenergic blocking drugs may lead to impaired metabolism, hyperglycemia, and insulin resistance due to the inhibition of EPI stimulation. There is an emerging interest in understanding how the biomarkers of AL and MetS are connected and influence each other. Current systematic reviews have concentrated on AL and health (42), health risk behaviors and AL (12), basal cortisol levels, and MetS (43). Also, chronic stress effects on glucocorticoids and catecholamines have been reported to be an influencing factor for MetS and CVDs (44). Thus, understanding the linkage between AL and MetS is of clinical relevance. Yet, the evidence for the association between the primary mediators of AL and MetS is limited. Thus, the main aim of the current systematic review is to critically examine the associations of the primary mediators of AL and MetS in the literature. In addition, the study aims to analyze these associations in a wide range of populations.

## 2 Methods

### 2.1 Study protocol

The current systematic review was conducted and reported based on the recommendations of the Preferred Reporting Items for Systematic Reviews and Meta-Analysis (PRISMA) (45). The completed PRISMA statement checklist is provided as a supplementary material (**Supplementary Tables 1, 2**).

### 2.2 Data source and search strategy

Two electronic databases, PubMed and Web of Science, were searched for articles published from January 2010 to December 2021 in English. The search strategy was based on the medical subject heading (MeSH) and non-MeSH search terms of keywords and the Boolean operators AND/OR ([Allostatic load; Allostatic overload; AL; Metabolic syndrome; MetS; Cortisol; Epinephrine; Norepinephrine; Dehydroepiandrosterone sulfate and DHEAS]). For additional information, the Cochrane library and the reference lists of systematic reviews found from the search were screened for related articles.

### 2.3 Eligibility criteria for study selection

The studies included in this systematic review met the following eligibility criteria: (I) observational studies (i.e., cross-sectional or case–control study) with an adult population (i.e., 18 years and above) that involved (II) study populations affected by MetS, obesity or overweight and control group; (III) studies examining the association between primary AL mediators: cortisol; epinephrine; norepinephrine; dehydroepiandrosterone sulfate and MetS, and (IV) original full-text studies in English. Exclusion criteria used in this systematic review were: (I) reviews, meta-analyses, case reports, expert opinions, trials, studies using animals or children, conference proceedings, and editorials, (II) duplication of the same data and population; and (III) studies using populations with other comorbidities except for individuals with MetS, overweight, or obesity. The Authors (FO and AB) established the search criteria for the study. The searches using the criteria established above for the selection of full-text articles were performed by one author (FO). Disagreements were resolved by a discussion with the second author (AB).

### 2.4 Data extraction

**Figure 1:**
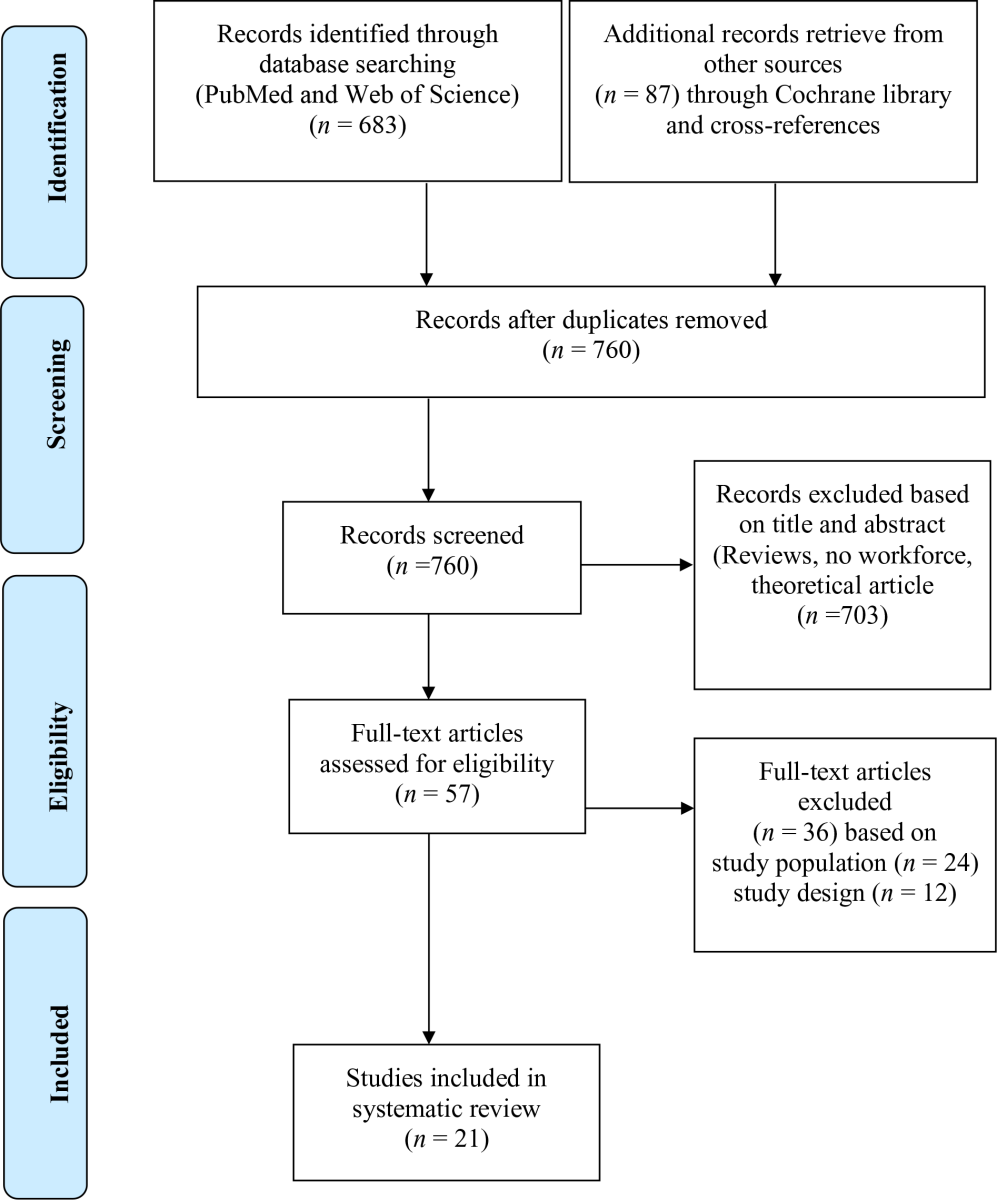
PRISMA flow diagram of search results.

The titles and abstracts of articles identified *via* the search were screened for relevance and cross-checked for eligibility. Full-text reports of relevant articles were also screened for their eligibility. Information on the search results is provided in **Figure 1**. Information from the included studies was extracted (see **Table 1** for more details). Data extraction was performed by one author (FO).

**Table 1:** Study characteristics of the association between the primary mediators of AL and MetS (*n* = 21).

Author/Date(Country)	MetS diagnosis	Sample		Primary ALmediators	Measurement techniques of primary AL mediators	Association between primary mediators of AL and MetS(Adjustment)	JBI Score
		N(sex %)	MetS (%)				
**1. Cross-sectional studies**							
Mazgelytė et al. (46) (Lithuania)	IDF	163 adults Men = 100%	MetS = 23.3 without MetS = 76.7	HCC	High-performance liquid chromatography	Significant association (*p <*0.005) was observed for higher HCC between participants with MetS (85.73 [150.88] ng/g) in comparison without MetS (36.50 [98.26] ng/g). (Non-adjusted).	8
				Serum cortisol	Enzyme-linked immunoassay	No significant association (*p* = 0.168) was observed for serum cortisol concentration between participants with MetS (221.78 [94.29] ng/ml) and participants without MetS (200.62 [128.15] ng/ml). (Non-adjusted).	
				Salivary cortisol	Enzyme-linked immunoassay	No significant association (p = 0.193) observed for salivary cortisol concentration between participants with MetS (9.16 [6.78] ng/ml) and participants without MetS (11.09 [9.85] ng/ml). (Non-adjusted).	
Lehrer et al. (47) (USA)	NCEP-ATP III (2004)	228 adults Men = 32% Women = 68%	Not applicable	HCC	Enzyme-linked immunoassay	Higher HCC was positively associated with MetS severity (*b* = 0.344, *SE* = 0.126, *95% CI* [0.106, 0.605]).(Adjusted for age, sex, race/ethnicity, income, medication use, physical activity, nervous and calm personality, hair washing, and bleach use).	7
Martins et al. (48) (Brazil)	NCEP-ATP III (2001)	80 adults Men = 43.7% Women = 56.3%	MetS = 50.0 without MetS = 50.0	Salivary cortisol	Radioimmunoassay	No significant association (*p* = 0.47) was observed for basal salivary cortisol between participants with MetS (44.4 ± 3.1 nmol/L) and participants without MetS (46.5 ± 2.9 nmol/L). (Non-adjusted).	5
Udenze et al. (49) (Nigeria)	NCEP-ATP III (2001)	100 adults Women = 100%	MetS = 50.0 without MetS = 50.0	Serum cortisol	Enzyme-linked immunoassay	No significant association (*p* = 0.437) was observed for serum cortisol between participants with MetS (12.80 ± 4.79 μg/dl) and participants without MetS (10.83 ± 6.59 μg/dl). (Non-adjusted).	5
Damgaard–Olesen et al. (50) (Denmark)	IDF	303 adults Men = 100%	Mets = 29.7 without MetS = 70.3	DHEAS	TurboFlow-Liquid Chromatography-Mass Spectrometry LC-MS/MS	No significant association (*p* = 0.23) was observed for DHEAS between participants with MetS (*Geometric Mean* = 4,527 nmol/L) and participants without MetS (*Geometric Mean* = 4,185 nmol/L). (Non-adjusted).	8
Constantinopoulos et al. (51) (Greece)	IDF	37 adults. Men = 47% Women = 53%	MetS = 51.4 without MetS = 48.6	UFC	Chemiluminescence immunoassay	Significant association (*p >*0.01) was observed for higher 24-h UFC for participants with MetS (116.8 ± 106.6 μg/24-h) in comparison to participants without MetS (71.3 ± 62.7 μg/24-h). (Non-adjusted).	6
				Serum cortisol	Chemiluminescence immunoassay	Significant association (*p >*0.01) for higher serum cortisol was observed between participants with MetS (16.6 ± 7.2 μg/ml) in comparison to participants without MetS (10.7 ± 4.1 μg/ml). (Non-adjusted).	
				Salivary cortisol	Chemiluminescence immunoassay	Significant association (*p >*0.01) was observed for higher salivary cortisol between participants with MetS (0.87 ± 0.4 μg/ml) in comparison with participants without MetS (0.46 ± 0.21 μg/ml). (Non-adjusted).	
Corbalán-Tutau et al. (52) (Spain)	IDF	70 adults Women = 100%	MetS = 57.0 without MetS = 43.0	Salivary Cortisol	Radioimmunoassay	Significant associations (*p <*0.05), in daily circadian markers for lower salivary cortisol levels (nmol/l) in participants with MetS in comparison participants without MetS. 8 am: MetS (17.1 ± 1.0 nmol/l) vs without MetS (25.3 ± 1.6 nmol/l). 14 pm: MetS (10.6 ± 0.3 nmol/l) vs without MetS (11.9 ± 0.4 nmol/l). 23 pm: MetS (5.0 ± 0.2 nmol/l) vs without MetS (6.3 ± 0.3 nmol/l) (Non-adjusted).	8
Almadi et al. (53) (Australia)	IDF	204 adults Men = 100%	MetS = 31.9 without MetS = 68.1	Salivary Cortisol	Electrochemiluminescence	Significant association (*p <*0.05) was observed for higher salivary cortisol between stress group with MetS (326.9 ± 153.3 nmol/L) in comparison with non-stress group without MetS (267.3 ± 99.2 nmol/L). (Adjusted for age, type of work, physical activity, awakening time, and work overcommitment).	8
Fabre et al. (54) (Belgium)	IDF & NCEP-ATP III (2001)	149 adults Men = 100%	MetS = 44.3 without MetS = 55.7	Serum Cortisol	Chemiluminescence immunoassay	No significant association (*p >*0.05) was observed for serum cortisol between participants with MetS (13.7 [5.7–23.6] μg/dl) and participants without MetS (13.3 [5.9–29.4] μg/dl). (Adjusted for age and BMI).	8
**Mattei et al. (55) (USA)	AHA/NHLBI	1318 adults Men = 27.8%Women = 72.2%	MetS = 67.6 without MetS = 32.4	UFC	Direct immunoenzymatic colorimetric method	No significant association (*p >*0.05) between UFC (mg/g creatinine) (OR = 1, 95% CI [0.995,1.004]) and participants with MetS. (Adjusted for age and sex).	8
				DHEAS	Electrochemiluminescence	No significant association (*p >*0.05) was observed for DHEAS (OR = 1, 95% CI [1,1] ng/ml) and MetS. (Adjusted for age and sex).	
				Urinary EPI	Direct immunoenzymatic colorimetric method.	No significant association (*p >*0.05) was observed between 12-h urinary EPI (µg/g creatinine) (OR = 0.97, 95% CI [0.938, 1.00]) and MetS. (Adjusted for age and sex).	
				Urinary NE	Direct immunoenzymatic colorimetric method.	No significant association (*p >*0.05) was observed between 12-h urinary NE (µg/g creatinine) (OR = 1, 95% CI [0.998, 1]) and MetS. (Adjusted for age and sex).	
Jang et al. (56) (Korea)	IDF	46 adults Men = 59%Women = 41%	MetS = 26.0 without MetS = 74.0	Salivary Cortisol	Competitive enzyme immunoassay	Significant association (*p* = 0.0001) was observed for higher midnight salivary cortisol levels between participants with MetS (70 ± 42.4 ng/dl) in comparison with participants without MetS (48.1 ± 36.8 ng/dl). (Non-adjusted).	8
Baudrand et al. (57) (Chile)	NCEP-ATP III (2004)	221 adults Men = 26.2%Women =73.8%	MetS = 58.8 without MetS = 41.2	UFC	High-performance liquid Chromatography (HPLC)	No significant association (*p* = 0.196) was observed for UFC between participants with MetS (21.13 [11.3–28.1 µg/24 h]) and participants without MetS (24.81 [13.8–31.2 µg/24 h]). (Non-adjusted).	8
Esteghamati et al. (58) (Iran)	NCEP-ATP III (2001)	285 adults Men = 43.5%Women = 56.5%	MetS = 42.1 without MetS = 57.9	Serum cortisol	Radioimmunoassay	No significance association (*p >*0.05) was observed for serum cortisol between males and females with MetS (15.16 ± 5.04 µg/dl) and with males and females without MetS (14.56 ± 4.66 µg/dl). (Non-adjusted). Significant association (*p <*0.05) for higher serum cortisol in males with MetS (17.74 ± 5.1 µg/dl). (Adjusted for age, WC, and BMI).	6
Park et al. (59) (Korea)	NCEP-ATP III (2004)	1881 adults Men = 43.9%Women = 56.1%	Mets = 27.3 without MetS = 72.7	Serum Cortisol	Radioimmunoassay	Significant association was observed for both males (*b* = 1.084, SE = 0.021, *p* = 0.000) and females (*b* = 1.031, SE = 0.015, *p* = 0.040) with higher serum cortisol (μg/dl) and MetS. (Adjusted for age and BMI).	6
Austin-Ketch et al. (60) (USA)	NCEP-ATP III (2001)	102 adults Men = 59.8%Women = 40.2%	MetS = 17.7 without MetS = 82.3	Salivary Cortisol	Chemiluminescence immunoassay	No significant association (*p* = 0.930) was observed for salivary cortisol and the presence of MetS (F [2, 63] = 0.072; partial η (= 0.002). (Non-adjusted) Significance difference (*p* = 0.05) was observed in mean diurnal AUC values between males with MetS and males without MetS. (Non-adjusted).	8
Bengtsson et al. (61) (Sweden)	NCEP-ATP III (2001)	175 adults Men = 48%Women = 52%	MetS = 16.6 without MetS = 83.4	Salivary Cortisol	Radioimmunoassay	Significant association (*p* = 0.02) was observed for higher salivary cortisol awakening response percentage (CAR%) for women with MetS (CAR% = 91.4 [17.0 nmol/L] in comparison to men without MetS (CAR% = 38.5[13.1nmol/L]. (Non-adjusted).	8
62 (Taiwan)	AHA/NHLBI	585 adults Men = 100%	MetS = 33.3 without MetS = 66.7	DHEAS	Electrochemiluminescence	Significant *(p >*0.001) association was observed for higher DHEAS between participants with MetS (3.1 ± 2.0 µmol/L) in comparison with participants without MetS (2.4 ± 1.6 µmol/L). (Non-adjusted).	8
Phillips et al. (63) (United Kingdom)	IDF	4255 adults Men = 100%	MetS = 13.7 without MetS = 86.3	Serum cortisol	Radioimmunoassay	No significant association between serum cortisol and MetS was observed (OR = 1.31; 95%CI: 0.98, 1.76; *p* = 0.07). (Adjusted for age, place of service, ethnicity, marital status, alcohol consumption, smoking, household income and education grade).	8
				DHEAS	Radioimmunoassay	Higher DHEAS concentrations significantly reduced MetS (OR = 0.56, 95% CI 0.46–0.69, *p <*0.001). (Adjusted for age, place of service, ethnicity, marital status, alcohol consumption, smoking, household income and education grade).	
**2. Case–control studies**							
Garcez et al. (64) (Brazil)	JIS	250 adults Women = 100%	MetS = 20.0 Controls = 80.0	Salivary Cortisol	Chemiluminescence immunoassay	No significant associations were observed for daily circadian cortisol changes between participants with MetS and participants without MetS. awakening cortisol levels: MetS (5.37 ± 4.10 nmol/l) vs without MetS (6.03 ± 5.39 nmol/l, *p* = 0.57), salivary cortisol levels after work: MetS (2.78 ± 2.87 nmol/l) vs without MetS (2.78 ± 2.85 nmol/l, *p* = 0.93). (Adjusted for age).	9
Kazakou et al. (65) (Greece)	AHA/NHLBI	I59 adults Men = 42.1% Women = 57.9%	MetS = 54.1 Controls = 45.9	Serum cortisol	Chemiluminescence immunoassay	No significant association (*p >*0.05) was observed for serum cortisol between participants with MetS (466.27 ± 146.23 nmol/L) and participants without MetS (455.24 ± 168.30 nmol/L). (Non-adjusted).	9
Özçelik et al. (66) (Turkey)	NCEP-ATP III (2001)	55 adults. Women = 100%	MetS = 63.6 Controls = 36.4	UFC	Immunoenzymatic colorimetric method	Significant association (*p <*0.05) was observed for lower serum DHEAS between participants with MetS (116 [68.00–152.00] µg/dl) in comparison without MetS (166.50[138.00–213.75 µg/dl]). (Non-adjusted).	7
				Serum cortisol	Immunoenzymatic colorimetric method	Significant association (*p <*0.001) was observed for higher serum cortisol between participants with MetS (18.77 [9.60–25.41] µg/dl) in comparison with participants without MetS (12.71 [11.29–15.70] µg/dl). (Non-adjusted).	
				DHEAS	Electrochemiluminescence	Significant association (*p <*0.05) was observed for lower DHEAS between participants with MetS (116 [68.00–152.00] µg/dl) in comparison without MetS (166.50 [138.00–213.75] µg/dl). (Non-adjusted).	

*Key: IDF, International Diabetes Federation; AHA/NHLBI, American Heart Association/National Heart, Lung, and Blood Institute; MetS, Metabolic syndrome; DHEAS, Dehydroepiandrosterone sulfate; NCEP-ATP III, National Cholesterol Education Program’s Adult Treatment Panel III; UFC, Urinary free cortisol; CAR%, cortisol awakening response percentage; JIS, Joint Interim Statement; HCC, hair cortisol concentrations; SC, Serum cortisol; BMI, Body mass index; WC, Waist circumference; JBI, The Joanna Briggs checklist for analytical cross-section studies and case–control studies.

**Additional data was obtained from the Authors.

### 2.5 Assessment of study methodological quality

The Joanna Briggs Institute (JBI) critical appraisal tool was used to assess the methodological quality of the included studies (67). The questions in the JBI included: (a) a clear description of study objectives; (b) clear description of inclusion and exclusion criteria for study participants; (c) a clear description of the population; (d) clearly describing the method of measurement of exposure; (e) characteristics of the mediator/moderator and outcome variables reported; (f) identifying and measuring potential confounders; (g) control of confounders; and (h) appropriate statistics used in answering study objectives. The JBI score assigns a maximum of 8 points (for cross-sectional studies) and 10 points (for case–control studies), indicating the highest study quality. For this systematic review, overall points of ≥5 for all cross-sectional and overall points of ≥6 for case–control studies were considered sufficient for inclusion. The studies were independently reviewed by one author (FO). This JBI tool has been used in other studies, making it a relevant tool to be used in this systematic review (68, 69).

### 2.6 Assessment of study quality control

The Strengthening the Reporting of Observational Studies in Epidemiology (STROBE) Checklist was used for study quality control assessment (70). The checklist contains a total of 22 items, which evaluated the reporting of each study’s title, abstract, introduction, methodology, results, and discussion. One author (FO) evaluated the studies for each item on the STROBE checklist as “yes,” “no,” or “not applicable” and calculated the number and percentage (%) of the included studies matching each item on the STROBE checklist. The completeness of reporting (COR) was calculated from the formula: COR (%) = (yes ÷ (yes + no) × 100) for each included study. A COR score of (if 0%–49% of items were met) was considered low, (if 50%–74% of items were met) was considered “moderate,” and (if ≥75% of items were met) was considered “high.” A similar protocol has been used in a study published elsewhere (12).

### 2.7 Statistical methods

All studies derived from the two databases that provided data on primary mediators of AL and MetS were considered eligible for analysis using Microsoft Excel version 16.63.1 (Microsoft Corporation, Redmond-Washington, USA). The included studies reported the associations between primary mediators of AL and MetS, usually using descriptive statistics (i.e., means and standard deviations) and inferential statistical models. Descriptive statistics, mainly frequency distributions, were used to report all the pooled measurements of the primary mediators of AL (i.e., salivary cortisol, serum cortisol, urinary cortisol (UFC), hair cortisol concentration (HCC), DHEAS, urinary EPI, and urinary NE) and their association with MetS.

## 3 Results

### 3.1 Main characteristics of studies included

The search of the databases (PubMed, *n* = 173 and Web of Science, *n* = 510) yielded 683 studies. Additional records retrieved from other sources through the Cochrane library and cross-references yielded 87 studies, resulting in an overall 770 studies. Out of these studies, 57 studies were assessed for eligibility after excluding 703 studies. Only 21 studies were considered for this systematic review after excluding 24 studies based on the study population and 21 studies based on study design. The included studies had a total number of participants (*n* = 10,666) with ages between 18 and 75 years. The sample size ranged from 37 to 4,225 participants within different populations (i.e., MetS, without MetS, workers, veterans, overweight, and obese). The included studies were published on different continents, consisting of: Europe (*n* = 8), Asia (*n* = 5), North America (*n* = 3), South America (*n* = 3), Africa (*n* = 1), and Australia (*n* = 1). Eighteen studies were cross-sectional, and three were case–control studies. Studies that reported cortisol as the primary mediator of AL were grouped into long-term cortisol measures (i.e., urinary cortisol [UFC] and hair cortisol concentration [HCC]) and short-term cortisol measures (i.e., salivary and serum cortisol). From the included studies, four studies measured UFC, two studies measured HCC, nine studies measured salivary cortisol, and nine studies measured serum cortisol. DHEAS was measured in six studies as a primary mediator of AL. Urinary EPI and urinary NE were measured in one study as primary mediators of AL (see **Table 1** for details).

### 3.2 Main results

The results are reported based on the different primary mediators of AL and its association with MetS. Afterwards, the results are summarized with the findings. Two studies (55, 57) found no significant associations, whereas two studies (51, 66) found significant associations between UFC and MetS. Two studies (46, 47) found significant associations between HCC and MetS. Four studies (46, 48, 60, 64) found no significant association, whilst six studies (51–53, 56, 60, 61) found significant associations between salivary cortisol and MetS. Six studies (46, 49, 54, 58, 63, 65) found no significant associations, but four studies (51, 58, 59, 66) found significant associations between serum cortisol and MetS. Two studies (50, 55) found no significant associations, while three studies (62, 63, 66) found significant associations between DHEAS and MetS. One study (55) found no significant associations between urinary EPI, urinary NE, and MetS.

### 3.3 Summary of results

Regarding cortisol, it can be summarized that UFC (12-h or 24-h) showed a significant association with MetS in 50% of the studies, and HCC showed a significant association with MetS in 100% of the studies. Short-term measures including serum cortisol showed a significant association with MetS in 40% of the studies, and salivary cortisol showed a significant association with MetS in 60% of the studies, respectively. In 60% of the studies, DHEAS showed a significant association with MetS. Both urinary EPI and NE (12-h) showed no significant association with MetS in 100% of the studies.

## 4 Assessment heterogeneity

### 4.1 Metabolic syndrome diagnoses criteria

There were variations in the diagnosis of MetS in the included studies. Six studies used the “Third National Cholesterol Education Program and Adult Treatment Panel” (NCEP-ATP III) 2001 criteria, and three studies used the 2004 criteria. Seven studies used the “International Diabetes Federation” (IDF) criteria. Three studies used the 2005 criteria of the “American Heart Association/National Heart, Lung, and Blood Institute (AHA/NHLBI). One study used the “Joint Interim Statement” (JIS) criteria. One study used both IDF and NCEP-ATP III (2001) criteria. The different institutional criteria used in the diagnosis of MetS are explained in detail in a study by Alberti et al. (2).

### 4.2 Assessment criteria for primary allostatic load markers: Measurement of cortisol

UFC (12-h or 24-h) was measured in four studies with chemiluminescence immunoassay (51), direct immunoenzymatic colorimetric method (55), high-performance chromatography (57), and electrochemiluminescence immunoassay (66). HCC was measured in two studies using enzyme-linked immunoassay (47) and high-performance chromatography (46). Salivary cortisol was measured in nine studies with enzyme-linked immunoassay (46), chemiluminescence immunoassay (51, 60, 64), radioimmunoassay (RIA) (48, 52, 61), electrochemiluminescence immunoassay (53), and competitive enzyme immunoassay (56). Serum cortisol was measured in eight studies with enzyme-linked immunoassay (46, 49, 51, 54, 65), RIA (58, 59), and electrochemiluminescent immunoassay (66).

### 4.3 Assessment criteria for primary allostatic load markers: Measurement of DHEAS

DHEAS was measured in five studies with the turboFlow-LC-MS/MS method (50), chemiluminescent immunoassay (55, 66), electrochemiluminescent immunoassay (62), and RIA (63).

### 4.4 Assessment criteria for primary allostatic load markers: Measurement of epinephrine and norepinephrine

In one study, urinary EPI and urinary NE (12-h) were measured using a 2-CAT enzyme immunoassay read on a Dynex MRX 96-well plate reader (55).

### 4.5 Study methodological quality

Applying the JBI tool, twenty studies representing 95.2% were judged very well to excellent (≥6 to ≥10) while one study representing 4.8% was judged fairly good (≥5). The summary of scores of the included studies is presented in **Tables 2**, **3**.

**Table 2:** Joanna Briggs Institute (JBI) scores for cross-sectional studies.

Study	Participants and setting described in detail,including similarity of controls	Criteria for inclusion clearly defined and exposures similarly measured	Exposure measured invalid and reliable way	Objective, standard criteria used for measurement of condition	Confounding factors identified	Strategies to deal with confounding factors stated	Outcomes measured invalid and reliable way	Appropriatestatistical analysis used?
Mazgelytė et al. (46) (Lithuania)	+	+	+	+	+	+	+	+
Lehrer et al. (47) (USA)	+	+	+	−	+	+	+	+
Martins et al. (48) (Brazil)	+	−	+	+	−	−	+	+
Udenze et al. (49) (Nigeria)	+	+	+	+	−	−	+	+
Damgaard-Olesen et al. (50) (Denmark)	+	+	+	+	+	+	+	+
Constantinopoulos et al. (51) (Greece)	+	+	+	+	−	−	+	+
Corbalán-Tutau et al. (52) (Spain)	+	+	+	+	+	+	+	+
Almadi et al. (53) (Australia)	+	+	+	+	+	+	+	+
Fabre et al. (54) (Argentina)	+	+	+	+	+	+	+	+
Mattei et al. (55) (USA)	+	+	+	+	+	+	+	+
Jang et al. (56) (Korea)	+	+	+	+	+	+	+	+
Baudrand et al. (57) (Chile)	+	+	+	+	+	+	+	+
Esteghamati et al. (58) (Iran)	+	+	+	+	−	−	+	+
Park etal. (59) (Korea)	+	+	+	+	−	−	+	+
Austin-Ketch et al. (60) (USA)	+	+	+	+	+	+	+	+
Bengtsson et al. (61) (Sweden)	+	+	+	+	+	+	+	+
Chen et al. (62) (Taiwan)	+	+	+	+	+	+	+	+
Phillips et al. (63) (United Kingdom)	+	+	+	+	+	+	+	+

**Table 3:** Joanna Briggs Institute (JBI) scores for Case–control studies.

Study	Group comparable in the presence of disease in cases and absence of diseases in controls	Cases and controls matched appropriately	Same criteria used for identifying cases and controls	Exposure measured invalid and reliable way	Exposure measured in same way as cases and controls	Confounding factors identified	Strategies to deal with confounding factors stated	Outcomes measured in standard, valid and reliable way for cases and controls	Exposure period of interest long enough to be meaningful	Appropriate statistical analysis used?
Garcez etal. (64) (Brazil)	+	+	+	+	+	+	+	+	–	+
Kazakou etal. (65) (Greece)	+	+	+	+	+	+	+	+	–	+
Özçelik etal. (66) (Turkey)	+	+	+	+	+	−	−	+	–	+

### 4.6 Study quality control

The STROBE Checklist for study quality control assessment was performed on the 21 included studies. From the included studies, one study had moderate score (COR = 50%–74%), and twenty studies had high score (COR = ≥75%). The mean COR score for the included studies was 87.0 ± 6.4% suggesting a higher study quality control (see **Table 4**).

**Table 4:** STROBE Statement—A checklist of items and the completeness of reporting score (COR %) for the included studies (*n =* 21).

	Item No.	Recommendation	Criteria Met (*N*, %) Yes No N/A		
**Title and abstract**	1	(*a*) Indicate the study’s design with a commonly used term in the title or the abstract	21(100)	0 (0)	0 (0)
		(*b*) Provide in the abstract an informative and balanced summary of what was done and what was found	21 (100)	0 (0)	0 (0)
**Introduction**					
Background/rationale	2	Explain the scientific background and rationale for the investigation being reported	21 (100)	0 (0)	0 (0)
Objectives	3	State-specific objectives, including any prespecified hypotheses	21 (100)	0 (0)	0 (0)
**Methods**					
Study design	4	Present key elements of study design early in the paper	21 (100)	0 (0)	0 (0)
Setting	5	Describe the setting, locations, and relevant dates, including periods of recruitment, exposure, follow-up, and data collection	21 (100)	0 (0)	0 (0)
Participants	6	(*a*) *Cohort study*—Give the eligibility criteria, and the sources and methods of selection of participants. Describe methods of follow-up *Case–control study*—Give the eligibility criteria, and the sources and methods of case ascertainment and control selection. Give the rationale for the choice of cases and controls *Cross-sectional study*—Give the eligibility criteria, and the sources and methods of selection of participants	N/A 3 (100) 16 (76.1)	N/A 0 (0) 2 (9.6)	N/A 0 (0) 3 (14.3)
		(*b*) *Cohort study*—For matched studies, give matching criteria and number of exposed and unexposed *Case–control study*—For matched studies, give matching criteria and the number of controls per case	3 (100)	0 (0)	0 (0)
Variables	7	Clearly define all outcomes, exposures, predictors, potential confounders, and effect modifiers. Give diagnostic criteria, if applicable	19 (90.4)	2 (9.6)	0(0)
Data sources/measurement	8*	For each variable of interest, give sources of data and details of methods of assessment (measurement). Describe comparability of assessment methods if there is more than one group	21 (100)	0 (0)	0 (0)
Bias	9	Describe any efforts to address potential sources of bias	19(90.4)	2 (9.6)	0 (0)
Study size	10	Explain how the study size was arrived at	20 (95.2)	1 (4.8)	0 (0)
Quantitative variables	11	Explain how quantitative variables were handled in the analyses. If applicable, describe which groupings were chosen and why	21 (100)	0 (0)	0 (0)
Statistical methods	12	(*a*) Describe all statistical methods, including those used to control for confounding	20 (95.2)	1 (4.8)	0 (0)
		(*b*) Describe any methods used to examine subgroups and interactions	21 (100)	0 (0)	0 (0)
		(*c*) Explain how missing data were addressed	3 (14.3)	2 (9.6)	16 (76.1)
		(*d*) *Cohort study*—If applicable, explain how loss to follow-up was addressed *Case–control study*—If applicable, explain how matching of cases and controls was addressed *Cross-sectional study*—If applicable, describe analytical methods taking account of sampling strategy	20 (95.2)	1 (4.8)	0 (0)
		(*e*) Describe any sensitivity analyses	20 (95.2)	0 (0)	1 (4.8)
**Results**					
Participants	13*	(a) Report numbers of individuals at each stage of study—e.g., numbers potentially eligible, examined for eligibility, confirmed eligible, included in the study, completing follow-up, and analyzed	21 (100)	0 (0)	0 (0)
		(b) Give reasons for non-participation at each stage	5 (23.8)	3 (14.3)	13 (61.9)
		(c) Consider use of a flow diagram	0 (0)	21 (100)	0 (0)
Descriptive data	14*	(a) Give characteristics of study participants (e.g., demographic, clinical, social) and information on exposures and potential confounders	19 (90.4)	1 (4.8)	1 (4.8)
		(b) Indicate number of participants with missing data for each variable of interest	2 (9.6)	1 (4.8)	18 (85.6)
		(c) *Cohort study*—Summarize follow-up time (e.g., average and total amount)	N/A	N/A	N/A
Outcome data	15*	*Cohort study*—Report numbers of outcome events or summary measures over time	N/A	N/A	N/A
		*Case–control study—*Report numbers in each exposure category, or summary measures of exposure	3(100)	0 (0)	18 (0)
		*Cross-sectional study—*Report numbers of outcome events or summary measures	19 (90.4)	2 (9.6)	0 (0)
Main results	16	(*a*) Give unadjusted estimates and, if applicable, confounder-adjusted estimates and their precision (e.g., 95% confidence interval). Make clear which confounders were adjusted for and why they were included	19 (90.4)	2 (9.6)	0 (0)
		(*b*) Report category boundaries when continuous variables were categorized	13 (61.9)	1 (4.8)	7 (33.3)
		(*c*) If relevant, consider translating estimates of relative risk into absolute risk for a meaningful time period	13 (61.9)	5 (23.8)	3 (14.3)
Other analyses	17	Report other analyses done—e.g., analyses of subgroups and interactions, and sensitivity analyses	21 (100)	0 (0)	0 (0)
**Discussion**					
Key results	18	Summarize key results with reference to study objectives	21 (100)	0 (0)	0 (0)
Limitations	19	Discuss limitations of the study, taking into account sources of potential bias or imprecision. Discuss both direction and magnitude of any potential bias	11 (52.4)	10 (47.6)	0 (0)
Interpretation	20	Give a cautious overall interpretation of results considering objectives, limitations, multiplicity of analyses, results from similar studies, and other relevant evidence	15 (71.4)	6 (28.6)	0 (0)
Generalisability	21	Discuss the generalizability (external validity) of the study results	9 (42.9)	12 (57.1)	0 (0)
**Other information**					
Funding	22	Give the source of funding and the role of the funders for the present study and, if applicable, for the original study on which the present article is based	13 (61.9)	7 (33.3)	1 (4.8)
**Completeness of Reporting mean of the 21 studies (%)**			**87.0 ± 6.4%**		

"*" refer to Vandenbroucke et al., (71).

## 5 Discussion

This systematic review examines the association between primary mediators of AL and the presence of MetS. The systematic review further highlights psychosocial, environmental, anthropometric, and socio-demographic factors influencing the association between the primary mediators of AL and MetS. Regarding the primary AL mediator cortisol, it is to be noted that MetS is associated with higher HCC and in some studies further with UFC, serum cortisol and salivary cortisol. In addition, the other HPA axis-related marker, DHEAS, showed a significant association with MetS. On the other hand, regarding primary mediators of the autonomic nervous system, there is no significant association between urinary EPI, urinary NE, and MetS. The findings of the current systematic review demonstrate that chronic stress leading to higher cortisol levels and low DHEAS levels may be associated with a hyperresponsive HPA axis. In the pathogenesis of MetS, this occurs.

Also, the two studies (51, 66) that reported an association between UFC and MetS involved participants with a body mass index (BMI = 39.3–52.4 kg/m^2^). In contrast, the other two studies (55, 57) that reported no association between UFC and MetS had participants with a BMI of 29.2–32.9 kg/m^2^. The results indicate that adults with higher BMI or obesity are most likely to have MetS and a hyperresponsive HPA axis due to increased cortisol levels. The results confirm a previous systematic review that reported that obesity appears to be related to a hyperresponsive HPA axis (72). An increase in body weight may lead to chronic low-grade inflammation, which may provoke an increased production of pro-inflammatory cytokines. The increased production of pro-inflammatory cytokines may cause chronic HPA axis activation, leading to visceral obesity and MetS (73). The discrepancies in the findings on the association between UFC and MetS in this systematic review may be attributed to the ethnicity variation in the diagnosis of MetS. This may be due to the varying measurement techniques employed in the various studies. Alberti et al. (2) reported different ethnicity variations in the diagnosis of MetS. Also, none of the included studies used the gold standard in measuring UFC, i.e., 24-h UFC measured by liquid chromatography with tandem mass spectrometry (LC-MS/MS) (74). Hence, longitudinal research focusing on the gold standard for measuring UFC and its association with MetS across different ethnicities is vital for understanding chronic stress’s effects on metabolic abnormalities.

The literature review showed inconsistent findings based on sex for the association between salivary cortisol and serum cortisol with MetS. Similar findings based on cortisol and sex have been reported in another systematic review (72). Significant associations between higher serum cortisol (59) and higher cortisol awakening response (CAR) (61) were found for both men and women with MetS. Bengtsson et al. (61) further reported an association between CAR and depressive symptoms in women. CAR is the measure of the dynamics of the HPA axis response upon awakening (75). A dampened CAR shows impaired HPA axis reactivity and has been suggested to be associated with metabolic abnormalities (75, 76). On the contrary, Esteghmati et al. (58) found only an association between serum cortisol and MetS in men.This shows that cortisol is a key marker in the stress response in both men and women. This calls for future research to study stress effects on HPA axis dysregulation and metabolic abnormalities in both sexes. Additionally, the literature review found mixed findings for the association between salivary and serum cortisol and MetS in workers. The studies in poultry workers (64) and police officers (60) found no association between salivary cortisol and MetS. In contrast, Almadi et al. (53) found associations between salivary cortisol and MetS in different workers (i.e., veterinary, agricultural, textile, and poultry industries). Also, the only study (63) that measured serum cortisol in veterans of the Vietnam-era USA army found no association with MetS. Notably, a previous systematic review reported that the effects of job strain and MetS appear to be significant (77). This shows that different job strain may affect the neuroendocrine systems differently in the pathogenesis of MetS. Hence, workplace health promotion programs geared toward stress management are needed to prevent the adverse effects of job strain on the neuroendocrine system of workers (78).

In this systematic review, some studies (50, 55) reported no associations between DHEAS and MetS, while others did (62, 63, 66). Furthermore, Chen et al. (62) found that participants with MetS had a higher DHEAS (3.1 ± 2.0 µmol/L) as compared to participants without MetS (2.4 ± 1.6 µmol/L). This could be due to steroid biosynthetic defects of the adrenal glands or functional adrenal hyperplasia, and age-related changes in the adrenal secretory pattern of the participants (Age = 67.8 ± 8.4) employed in their study (79). DHEAS declines with age and may lead to age-specific diseases such as obesity and MetS (44). This age-related decline in DHEAS is attributed to a mechanism termed “adrenopause” (80). There are limited studies investigating the association between DHEAS and MetS. Hence, the interplay between DHEAS and MetS warrants further study.

The only study (55) that reported on urinary NE and urinary EPI found no significant association with MetS. Foremost, Zouhal et al. (40) demonstrated that increased levels of catecholamines lead to lipolytic effects on visceral fats by β_1_- and β_2_-adrenoceptors. Conversely, β-adrenergic blocking drugs inhibit EPI stimulation, leading to impaired glucose metabolism, hyperglycemia, and insulin resistance (41). While most of the NE is secreted by the sympathetic nerve endings, the adrenal glands secrete EPI (81). Thus, these catecholamines, which play roles under stress conditions to foster thermogenesis and secretion of insulin, may operate in a divergent fashion in the pathogeneses of MetS (41). From a research perspective, measuring 12-h urine collections for EPI and NE may be labor-intensive and impractical due to poor adherence (14). These findings should be interpreted with caution due to insufficient data.

Most studies used immunoassays for the measurement of cortisol. It should be noted that urine contains conjugated cortisol and other metabolites (82). Assessing UFC and salivary cortisol may lead to cross-reactivity of the antibodies in the immunoassays with other metabolites in urine and steroids in saliva (82, 83). Serum cortisol may not reflect the unbound (free) cortisol levels due to changes in albumin or cortisol binding globulin levels (74). Hence, using the LC-MS/MS to measure 24-h urinary cortisol is the gold standard (74). Mass spectrometry provides reliable cortisol measurement outcomes and prevents cross-reactivity of metabolites (74, 82, 84). Although Alberti et al. (2) released the joint interim statement concerning the diagnosis of MetS, only one study (64) used the joint interim statement criteria for the diagnosis of MetS in the included studies. Thus, caution should be taken when interpreting these results.

### 5.1 Strengths and limitations

This is the first systematic review to be conducted on the association between primary mediators of AL and MetS using cross-sectional and case–control studies. The large sample size and different populations in the included studies broaden the perspective on how the primary mediators of AL are associated with MetS. Despite these strengths, there are limitations to be reported. The cross-sectional data may prevent the cause–effect relationship between the primary mediators of AL and MetS at the time of measurement due to modifications of these mediators in the long term. The included studies had a wide difference in their methodologies. Most studies used different measurement techniques in measuring the primary mediators of AL, especially cortisol. This makes it difficult to make vivid comparisons and generalizations. Also, the included studies employed different institutional criteria for the diagnosis of MetS. This creates heterogeneity in the diagnosis of MetS. These factors could not be controlled in this systematic review. Additionally, only studies in English were included, which could have omitted potential studies published in other languages for inclusion.

## 6 Conclusion

The present systematic review revealed that there is a tendency for an association between higher UFC, HCC, serum cortisol, salivary cortisol, and lower DHEAS with MetS. There is no association between urinary NE and urinary EPI with MetS. Different assays for measuring the primary mediators of AL and the association of MetS may yield different outcomes. Research focusing on the standardization of measurement protocols for the primary mediators of AL would be vital for uniformity, comparability, and generalization. It is helpful to identify a cluster of biomarkers from the MetS diagnosis that best reflects the primary mediators of AL in order to foster preventive measures for individuals with altered levels of primary mediators. Future studies focusing on longitudinal data are warranted for clarification and understanding of the association between the primary mediators of AL and MetS.

## Data availability statement

The datasets generated for this study are available upon reasonable request to the corresponding author.

## Author contributions

FO and AB conceived the research question. FO wrote the first draft of the manuscript with the support of AB. FO, AB and P-MW discussed the results. All authors listed have made a substantial, direct, and intellectual contribution to the work and approved it for publication.

## Funding

The funder does not influence data collection, analysis, and interpretation or writing of the manuscript. The authors declare that they have no competing interests. The paper was funded by the Open Access Publishing Fund by the Deutsche Forschungsgemeinschaft (DFG, German Research Foundation)-Projektnummer 491466077. The present study was supported by a scholarship from DAAD (grant-number: 57552340) to Francis Osei.

## Conflict of interest

The authors declare that the research was conducted in the absence of any commercial or financial relationships that could be construed as a potential conflict of interest.

## Publisher’s note

All claims expressed in this article are solely those of the authors and do not necessarily represent those of their affiliated organizations, or those of the publisher, the editors and the reviewers. Any product that may be evaluated in this article, or claim that may be made by its manufacturer, is not guaranteed or endorsed by the publisher.
